# Assortative Matching with Inequality in Voluntary Contribution Games

**DOI:** 10.1007/s10614-017-9774-5

**Published:** 2017-11-18

**Authors:** Stefano Duca, Dirk Helbing, Heinrich H. Nax

**Affiliations:** 1 Department of Humanities, Political and Social Sciences, ETH Zürich, Clausiusstr. 50, 8092 Zurich, Switzerland; 2 Department of Humanities, Political and Social Sciences, ETH Zürich, Clausiusstr. 37, 8092 Zurich, Switzerland

**Keywords:** Assortative matching, Public goods, Heterogeneity, Inequality, Computational economics

## Abstract

Voluntary contribution games are a classic social dilemma in which the individually dominant strategies result in a poor performance of the population. However, the negative zero-contribution predictions from these types of social dilemma situations give way to more positive (near-)efficient ones when assortativity, instead of random mixing, governs the matching process in the population. Under assortative matching, agents contribute more than what would otherwise be strategically rational in order to be matched with others doing likewise. An open question has been the robustness of such predictions when heterogeneity in budgets amongst individuals is allowed. Here, we show analytically that the consequences of permitting heterogeneity depend crucially on the exact nature of the underlying public-good provision efficacy, but generally are rather devastating. Using computational methods, we quantify the loss resulting from heterogeneity vis-a-vis the homogeneous case as a function of (i) the public-good provision efficacy and (ii) the population inequality.

**Electronic supplementary material:**

The online version of this article (10.1007/s10614-017-9774-5) contains supplementary material, which is available to authorized users.

## Introduction

Suppose a population of agents faces the *collective action* (Olson 1965) challenge to provide public goods by means of simultaneous, separate *voluntary contributions games* (Isaac et al. 1985). In each one, the collective would benefit from high contributions but individuals may have strategic incentives (Nash 1950) to contribute less. Such situations, also known as ‘social dilemmas’, are related to collective management of ‘common-pool resources’ (Ostrom 1990; Schlager and Ostrom 1992) and often result in underprovisioning of the public good (i.e. tragedy of the commons as in Hardin 1968) because of the misalignment of collective interests and strategic incentives.

Generally, grave underprovision of the public good is the unique Nash equilibrium when individual contribution decisions are independent of the matching process. Andreoni (1988)’s model of a linear public goods game with random re-matching of groups is the best-known experimental instantiation of this, and numerous studies have reported corresponding decays in contributions when such games are played in the laboratory (Ledyard 1995; Chaudhuri 2011). Predictions may change dramatically, however, when agents are matched ‘assortatively’ instead, that is, based on their pre-committed choice on how much to contribute so that high (low) contributors are matched with other high (low) contributors. Such mechanisms have been coined ‘meritocratic group-based matching’ (Gunnthorsdottir et al. 2010a), short ‘meritocratic matching’ (Nax et al. 2014).^1^ Under meritocratic matching, new equilibria emerge through assortative matching that are as good as near-efficient (Gunnthorsdottir et al. 2010a; Nax et al. 2014). Indeed, when better (i.e. more efficient) equilibria exist, humans have been shown to consistently play them in controlled laboratory environments (Gunnthorsdottir et al. 2010a, b; Nax et al. 2017; Rabanal and Rabanal 2014).

In this paper, we address the important question of how robust the positive predictions stemming from assortative matching are. To assess this, we generalize the baseline model on two dimensions. On the one hand, we consider a range of public-goods provision efficacies that nests the standard marginal-per-capita-rate-of-return (‘mpcr’) model as a special, linear case. On the other hand, we allow heterogeneity in players’ budgets, expressing the *ex ante* inequality amongst individuals. In other contexts, heterogeneity has been shown to ‘help’ cooperation (Perc 2011). Our work, in particular, builds on one prior attempt at generalizing the standard model in terms of heterogeneity by Gunnthorsdottir et al. (2010b), who consider two levels of budgets in the standard case of mpcr-linear payoffs.

Methodologically, we blend analytical and computational approaches. Our results summarize as follows. We show analytically that the consequences of permitting heterogeneity in terms of provision of the public good depend crucially on the exact nature of the underlying public-good provision efficacy, but generally are devastating. Indeed, all near-efficient Nash equilibria that exist under homogeneity fall apart when heterogeneity is allowed. Instead, we are either back at the negative all-contribute-nothing equilibrium or new, previously impossible, complex mixed-strategy Nash equilibria emerge. In the latter case, the expected level of resulting public-good provision depends crucially on (i) the public-good provision efficacy and (ii) the population inequality. These mixed equilibria are virtually impossible to characterize and to evaluate analytically for general cases. We therefore use computational methods and quantify the loss resulting from heterogeneity vis-a-vis the homogeneous case as a function of parameters regarding (i) and (ii). Thus, our analysis provides novel insights regarding the possible consequences in terms of making wrong predictions when assuming a homogeneous population, which in many real-world cases may be unrealistic.

The rest of this paper is structured as follows. Next, we set up the model including details about our computational algorithm. Section 3 contains the paper’s results. Section 4 concludes. An “Appendix” contains details of the analytical results.

## The Model

*N* players are assigned an initial endowment $w_i$, that might be different for each player *i*, and play the following game, of which all aspects are common knowledge:

1. *Actions* Each player makes a simultaneous and committed unilateral decision regarding how much of his endowment to contribute. We will indicate with $\alpha _i \in \left[ 0,1\right]$ the percentage of $w_i$ contributed by player *i*. We indicate with $\alpha = \left\{ \alpha _i \right\}$ the array of strategies obtained in this way and with $\alpha _{-i}$ the strategy vector obtained excluding agent *i*.
2. *Matching* Players are ranked by their effective contributions $s_i = w_i \alpha _i$ (from highest to lowest with random tie-breaking). They are then assigned to $M=\frac{N}{{{\mathcal {S}}}}$ equal-sized groups of size ${{\mathcal {S}}}$, such that the ${\mathcal {S}}$ highest-ranking players are assigned to the first group, the ${\mathcal {S}}$ second-highest ranking players are assigned to the second, etc.
3. *Outcome* Payoffs $\phi _i$ realize based on the contribution total in each group. Each player receives the amount that he did not contribute plus the sum of all the contributions made by the members of his group multiplied by a factor *Q* (the marginal-per-capita-rate-of-return):
  1 $$\begin{aligned} \phi _{i}\left( \alpha _{i}\mid \alpha _{-i}\right) =w_i \left( 1-\alpha _{i}\right) +Q\sum _{j\in G_{i}}\alpha _{j}^{\gamma }w_{j} \end{aligned}$$
  with $G_i$ being the group to which agent *i* belongs.

The mpcr *Q* represents the benefits of cooperation among members of the same group. When $\frac{1}{{\mathcal {S}}}< Q < 1$ the game is a social dilemma, meaning that group efficiency is maximized if every member contributes fully but doing so is always a dominated strategy.

The parameter $\gamma$ in the return from the common pool is a measure of the “goodness” of the public-good provision efficacy. For high values of $\gamma$ (super-linear payoffs) even a high percentage of contributions has little effect on the common pool, thus making the public good less fruitful. On the other end, for low $\gamma$ values (sub-linear payoffs) even a small contribution allows players to obtain large benefits from cooperation. It is important to notice that the value of $\gamma$ determines also the maximum *efficiency* 2 of the system ranging from $w_i +Q\sum _{j\in G_{i}}w_{j}$ for $\gamma \rightarrow 0$ to $w_i$ for $\gamma \rightarrow \infty$.

### Simulation

We simulate the above model in the following way:

**1.**: **Initialization: **We assign the initial endowments to each player sampling them from a truncated Gaussian distribution with mean $W_0$ and standard deviation $\sigma$. The distribution is truncated at $w_i=0$ and $w_i= 2W_0$ so that endowments are always positive and symmetrically distributed around $W_0$.
**2.**: **Setting initial strategies: **The initial strategy $\alpha _i$ of each player is set to 0, hence the simulation starts in the fully defective state.^3^
**3.**: **Dynamics of play: **Each player updates his strategy in the following way:

- With probability *p* he keeps playing the strategy played the round before.^4^
- With probability $1-p$ he myopically best responds to the strategies played by the other agents the round before:
  The agent checks what would his rank and consequent payoff be for each of the possible strategies^5^ he can play, given that the other agents keep playing the strategies played the round before. He then chooses the strategy that results in the highest payoff.

**4.**: **Group matching: **Based on the contribution of each player, groups are formed as described above and payoffs are then materialized.
**5.**: **Update and repeat: **The endowment of each player is then reset to his initial one and the algorithm repeats from step *3*.

After *T* rounds the algorithm stops and the population average of the strategies is computed. The procedure is repeated *EN* times and the ensemble average of the population average is obtained.

## Results

The above game exhibits different Nash equilibria depending on the value of $\gamma$ and on the players having different or the same initial endowments.

As already shown in Gunnthorsdottir et al. (2010a), in the case of linear payoff ($\gamma = 1$) and homogeneous players the voluntary contribution game with assortative matching has multiple pure strategy Nash Equilibria: one is non contribution by all players and the others are almost Pareto optimal equilibria in which nearly all players contribute their entire endowment and few (less than the group size) contribute nothing. It is easy to see (see “Appendix”) that this result actually holds for any value of $\gamma$ bigger than a threshold value $\bar{\gamma }$, with $\bar{\gamma } < 1$ and depending on groups size, mpcr and the total number of players. The only difference with the linear payoff case is that for $\gamma < 1$ the within group Nash Equilibrium is to contribute ${\bar{\alpha }} = \left( \frac{1}{Q \gamma }\right) ^{\frac{1}{\gamma -1}}$. Hence, for sublinear payoffs such that $\gamma > \bar{\gamma }$, the Nash Equilibria are: all players contribute ${\bar{\alpha }}$ and all players contribute their total endowment except for few players that contribute ${\bar{\alpha }}$. For very small values of the exponent of the public good provision, the numbers of non contributors^6^ in the near efficient equilibrium becomes too high to be sustained. Hence, even though the nearly efficient outcome would be highly rewarding, for $\gamma \le \bar{\gamma }$ the only existing equilibrium is that all players contribute ${\bar{\alpha }}$.^7^

A key property of the game, necessary for the nearly efficient equilibria to exist, is that ties in the ranking placement are broken at random. In all these equilibria, in fact, there exists a mixed group where fully contributive players are grouped with defectors, i.e. players contributing the within group Nash Equilibrium. In order for this to be a NE, the fully contributive players must have a sufficiently high probability to be grouped only with other full contributors so that they would not benefit (in expectation) by decreasing their contribution and be placed with certainty in the defectors group.

If each player is endowed with a different initial wealth, however, things change drastically. The heterogeneity of the players implies that there cannot be an equilibrium where more than one player contributes the same positive percentage of his endowment: Since players are ranked based on their effective contributions $s_i = w_i \alpha _i$, if more than one player were to contribute the same percentage, the one with the highest endowment would have a profitable deviation due to the existence of the mixed group. He could in fact contribute slightly more and be guaranteed to be placed in a better group. If the two players were already contributing everything, the wealthiest player could instead contribute slightly less and still be guaranteed to remain in the same group. For the above reason, the nearly efficient Nash Equilibria in which almost all players contribute everything does not exist for heterogeneous players. Moreover, any unique contribution $\alpha _i$ such that ${\bar{\alpha }}< \alpha _i < 1$ is also clearly not a Nash Equilibrium due to the fact that it would be possible to contribute less and still be placed in the same group.

Furthermore, for sublinear payoffs ($\gamma < 1$) the equilibrium in which all players contribute the within group Nash Equilibrium does not exist. Indeed, for $\gamma < 1$ we have that ${\bar{\alpha }}>0$ and if all players were to play ${\bar{\alpha }}$, players with a lower endowment would have a profitable deviation by increasing their contributions and being grouped with players with a higher endowment. Hence, for heterogeneous players and sublinear payoffs there exist no pure strategy Nash Equilibria for the game.

For superlinear payoffs ($\gamma \ge 1$), however, the within group Nash Equilibrium is to contribute nothing and hence the pure strategy equilibrium in which no player contributes anything continues to exist. Consequently, there are no mixed strategy Nash Equilibria for this game.

Table 1 summarizes which Nash Equilibria exist in which situation. In the “Appendix” we formally derive the results described in this section.

**Table 1:** In this table we show which equilibria exist for homogeneous and heterogeneous players depending on the value of $\gamma$

	${\gamma \le \bar{\gamma }}$			${\bar{\gamma }< \gamma <1}$			${\gamma \ge 1}$		
	PSL	MS	PSH	PSL	MS	PSH	PSL	MS	PSH
Homog.	✓	✗	✗	✓	✗	✓	✓	✗	✓
Heterog.	✗	✓	✗	✗	✓	✗	✓	✗	✗

For homogeneous players, for any payoff such that $\gamma$ is bigger than a threshold value $\bar{\gamma }$, there exist one Nash Equilibrium in which all players contribute nothing (indicated as PSL) and almost Pareto optimal Nash Equilibria where almost all players contribute everything and few (less than the group size) contribute the within group NE ${\bar{\alpha }}$. (PSH). For $\gamma < \bar{\gamma }$, the only Nash Equilibirum is for everybody to contribute ${\bar{\alpha }}$. In these cases, there exist no mixed strategy (MS) Nash Equilibrium. For heterogeneous players the situation is different for different values of $\gamma$. For sublinear payoffs ($\gamma < 1$) there exist no pure strategy equilibria and hence the only Nash Equilibrium is in mixed strategies. For superlinear payoffs ($\gamma \ge 1$), the only pure strategy Nash Equilibrium is non-contribution by all players

To obtain the mixed strategy equilibrium of the game we resort to computational methods. We simulate agents playing the game for different payoffs and different width of the initial wealth distribution.

We are mainly interested in a comparison between the (unique) equilibria in the case of heterogeneous players and the equilibria reached in the homogeneous case. In particular, we are interested in how much efficiency (see footnote 2) is lost due to the heterogeneity of players. Indeed, even though non contribution by all is a Nash Equilibrium, the quasi Pareto optimal equilibrium is payoff dominant^8^ and hence is the one to which we refer (when it exists). Furthermore, experimental results (Gunnthorsdottir et al. 2010a) have also shown that the nearly efficient equilibria are the one reached by the population.

**Fig. 1:**
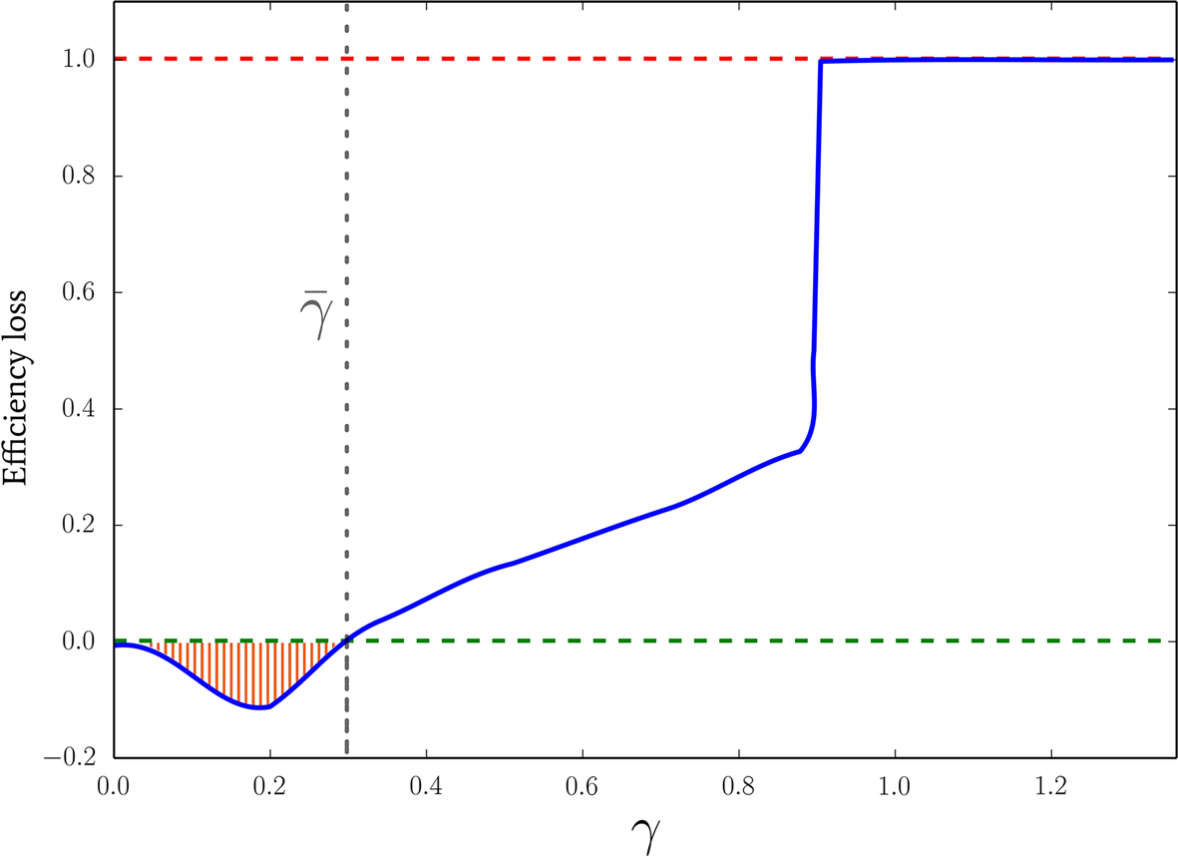
Here we show the loss of efficiency in case of heterogeneous players with respect to the level of contribution that would have been achieved in the homogeneous case. A $100\%$ loss (dashed red line) indicates that all players contribute nothing and thus that there is a complete loss of efficiency with respect to the homogeneous case. A $0\%$ loss (dashed green line) means that the system reaches the same efficiency that it would have reached in the case of homogeneous players. A negative loss (striped orange area below the green line) indicates that when endowments are heterogeneous the equilibrium reaches a higher efficiency than in the homogeneous case. As predicted, for superlinear payoffs $\gamma \ge 1$ the only possible equilibrium is non contribution by all and thus the loss of efficiency is total (right side of the picture). For intermediate sublinear payoff we can see that the efficiency is not completely lost and that it goes from being quite low to being closer to the homogeneous case. The quantitative value of efficiency that the mixed equilibrium achieves depends on the value of the mpcr and the width of the distribution of initial wealth as well as from other parameters. Finally, for $\gamma \le \bar{\gamma }$ we first observe a slight increase in efficiency (below the dashed green line) and then the efficiency approaches the homogeneous one (left side of the picture), on account of the benefits of cooperation being obtainable for an arbitrary small contribution. Hence, for a wide range of values of $\gamma$, we observe a significant loss in efficiency compared to the homogeneous case. The simulation was obtained for the following set of parameters: $N=100$, ${\mathcal {S}}=4$, $Q=0.6$, $W_0=2$, $EN = 50$, $p=0.2$ and $\sigma = 0.45$. For these parameters, $\bar{\gamma } \approx 0.29$. (Color figure online)

In Fig. 1 we plot the loss of efficiency due to the heterogeneity of the players with respect to the level of contributions that would have been achieved in the homogeneous case as a function of $\gamma$ and for a choice or representative parameters. A $100\%$ loss (dashed red line) indicates that all players contribute nothing and thus that there is a complete loss of efficiency with respect to the homogeneous case. A $0\%$ loss (dashed green line) means that the system reaches the same efficiency as it would with homogeneous players. A negative loss indicates that when endowments are heterogeneous the equilibrium reaches a higher efficiency than in the homogeneous case.

We observe, as predicted, that for superlinear payoffs the only possible equilibrium is non contribution by all and thus that the loss of efficiency is total. For intermediate sublinear payoff the system achieves different efficiency level, from quite low ones to levels closer to the homogeneous case. The quantitative value of efficiency that the mixed equilibrium achieves depends on the value of the mpcr and the width of the distribution of initial wealth as well as from other parameters. For values of the exponent of the public good provision lower than $\bar{\gamma }$, we initially observe an increase in efficiency when endowments are heterogeneous. This is due to the fact that for values of $\gamma$ slightly below $\bar{\gamma }$, the only equilibrium in the homogeneous case is to contribute ${\bar{\alpha }}$ but it would still be more efficient if more players contributed a higher percentage of their endowment. Finally, for $\gamma \rightarrow 0$, the heterogeneous system approaches the same efficiency of the homogeneous, on account of the benefits of cooperation being obtainable for an arbitrary small contribution.

Interestingly, one can observe that the efficiency loss doesn’t seem to change much for different widths of the endowment distribution (see online material).

For the width of the distribution equal to 0 we observe, as expected, the Nash Equilibria in case of homogeneous endowments.^9^

For different values of the marginal per capita rate of return we observe from the simulations (see online material for examples) that the higher the mpcr, the wider is the area with partial efficiency losses in the picture and the smaller is the gain in efficiency around $\bar{\gamma }$.

Hence we can conclude that for a wide range of payoffs, the more realistic assumption of heterogeneous players leads to a disruptive loss in efficiency when compared to the homogeneous case. For a very limited range of $\gamma$, however, heterogeneity seems to result in a small increase in efficiency.

## Summary of Results

In summary, mechanisms based on assortative matching promise large efficiency gains when the interaction is such that it is safe to assume that the population consists only of equals. In the presence of heterogeneity, however, whether and how much assortative matching is likely to gain the population (relative to random matching) depends crucially on the provision efficacy of the public good and on the precise degree of heterogeneity. This implies that guarantees of more equal playing fields in these environments may be as important as implementation of assortative matching.

### Electronic supplementary material

Below is the link to the electronic supplementary material.

Supplementary material 1 (pdf 232 KB)

Supplementary material 2 (pdf 231 KB)

Supplementary material 3 (pdf 118 KB)

## Appendix: Nash Equilibria

In this section we derive the Nash Equilibria of the generalized assortative matching voluntary contribution game.

We first define the game and derive some useful properties of it. Later, we show which equilibria exist for homogeneous and heterogeneous players for different values of the public good game efficacy.

*Notation*

- The expected payoff of player *i* is
  2 $$\begin{aligned} E_{i}\left( \alpha _{i},\alpha _{-i}\right) =\left( w_{i}-s_{i}\right) +\sum _{k=1}^{M}Pr\left( k\mid \alpha _{i},\alpha _{-i}\right) \cdot \left[ Q\cdot \left( S_{-i}^{k}+w_i\alpha _{i}^\gamma \right) \right] \end{aligned}$$
  with $M=\frac{N}{{\mathcal {S}}}$ the number of groups in the population, $S_{-i}^{k}$ the sum of the effective contributions in agent’s *i* group minus his own and $Pr\left( k\mid \alpha _{i},\alpha _{-i}\right)$ the probability of being ranked *k*th given $\alpha _i$ and $\alpha _{-i}$. We indicate with ${\mathcal {S}}$ the size of the groups.
- We say that players *i*, *j* are in class $C_r$ if $s_i = s_j = s^r$. We write $c_{r}\equiv \left| C_{r}\right| =D_{r}\cdot {\mathcal {S}}+\widetilde{c}_{r}$; $D_{r},\widetilde{c}_{r} \in N\cup \left\{ 0\right\}$.^10^ Note that by definition $0 \le \widetilde{c}_{r} < {\mathcal {S}}$.
- We call *h* the highest effective contribution and *H* the number of players s.t. $\alpha _i = h$; hence $H\equiv \left| C_{1}\right|$.
- We indicate with *z* the number of players playing the strategy ${\bar{\alpha }}$.
- Full heterogeneity means that $w_i \ne w_j \;\; \forall i\ne j$.

Let us first compute the within group best response.

### Lemma 1

The best response within a group is: $\alpha _i={\bar{\alpha }}$ if $0<\gamma <1$ and $\alpha _i = 0$ if $\gamma \ge 1$.

### Proof

The within group payoff is defined as following:

$$\begin{aligned} \phi _{i}\left( \alpha _{i}\mid \alpha _{-i}\right) =w_i\left( 1-\alpha _{i}\right) +Q\sum _{j\in G_{i}}\alpha _{j}^{\gamma }w_j \end{aligned}$$

with $G_i$ being the group to which agent *i* belongs and $\alpha _i \in \left[ 0,1\right]$.

The first order condition reads:

3 $$\begin{aligned} \frac{\partial \phi _{i}}{\partial \alpha _{i}}=-w_i+Qw_i\gamma \alpha _{j}^{\gamma -1}{\mathop {=}\limits ^{FOC}}0 \Rightarrow \alpha _i = \left( \frac{1}{Q\gamma }\right) ^{\frac{1}{\gamma -1}}\equiv {\bar{\alpha }} \end{aligned}$$

implying the following payoff for agent *i*:

4 $$\begin{aligned} w_i\left( 1-{\bar{\alpha }}\right) + w_iQ{\bar{\alpha }}^\gamma +C \end{aligned}$$

with *C* defined as $\sum _{j\in G_{i},j\ne i}\alpha _{j}^{\gamma }w_j$.

The payoff for the corner strategies instead is $w_i + C$ for $\alpha = 0$ and $w_iQ + C$ for $\alpha = 1$.

Since $0<Q<1$ the payoff for $\alpha = 0$ is always bigger than the one $\alpha = 1$. Now we need to check when $\phi _i\left( {\bar{\alpha }}\right) > \phi _i\left( 0\right)$ and hence when

$$\begin{aligned} Q\left( \frac{1}{Q\gamma }\right) ^{\frac{\gamma }{\gamma -1}}>\left( \frac{1}{Q\gamma }\right) ^{\frac{1}{\gamma -1}}. \end{aligned}$$

If $0< \gamma <1$ we can rewrite the above expression as $Q\left( Q\gamma \right) ^{\frac{\gamma }{1-\gamma }}>\left( Q\gamma \right) ^{\frac{1}{1-\gamma }}$ and hence as $\gamma ^\gamma > \gamma$; a condition that is always true for $0< \gamma <1$. If $\gamma >1$ we instead obtain the condition $\gamma ^{-\gamma } > \gamma ^{-1}$, never true for $\gamma >1$.

If $\gamma =0$, the FOC trivially results in $\alpha = 0$.

Hence, the best response for player *i* if the group placement is independent from $\alpha _i$ is $\alpha _i={\bar{\alpha }}$ for $0<\gamma <1$ and $\alpha _i = 0$ for $\gamma >1$. $\square$

### NE for Homogeneous Players

Here we compute what are the Nash Equilibria in the case of homogeneous players. The proof presented here follows closely the one in Gunnthorsdottir et al. (2010a), changing only to adapt it to the generalized payoff. Note that for homogeneous players we have $w_i = w \;\; \forall i$.

We first of all note that, for homogeneous players, all players playing the within group Nash Equilibria is a best response.

#### Lemma 2

$\alpha _i = {\bar{\alpha }}$ $\forall i$ is a Nash Equilibrium for every value of $\gamma$.

#### Proof

This is obviously an equilibrium. Since the mpcr *Q* is smaller than 1, there is no profitable deviation in being the only one contributing more than ${\bar{\alpha }}$. A player *i* deviating to a higher contribution would have the guarantee to be placed in the best group. The best group, however, would be such only because of him, thus making it not profitable to deviate. $\square$

In order to prove the existence of the high contribution equilibrium, we first observe that in order for the equilibrium to exist, the following conditions have to hold:

#### Lemma 3

If there are some strategies $\alpha _i$ s.t. $\alpha _i > {\bar{\alpha }}$, then in equilibrium we have to have $H > {\mathcal {S}}$ and $\left( H \; mod \; {\mathcal {S}} \right) > 0$. Hence a player contributing more than ${\bar{\alpha }}$ has a non-zero probability to be grouped with a player contributing less than him.

#### Proof

If the number of the highest contributors were a multiple of the group size, one of those player could unilaterally deviate and reduce his contribution by an amount small enough to remain in the same group and still profit from the deviation. For the same reason, that number of players has to be bigger than ${\mathcal {S}}$. If it were smaller, in fact, high contributors would benefit by deviating to ${\bar{\alpha }}$. $\square$

#### Lemma 4

If there are some strategies $\alpha _i$ s.t. $\alpha _i > {\bar{\alpha }}$, then the highest contribution *h* cannot be smaller than *w*. I.e. $\alpha _i = 1 \; \forall i \; \in C_1$.

#### Proof

We call $\alpha _h$ the strategy s.t. $\alpha _h w = h$. From Lemma 3 we know that (one of) the highest contributor(s) *i* has a non-zero probability to be grouped with some other player contributing less. Hence, if agent *i* were playing $\alpha _h < 1$, he could increase his contribution by an arbitrary small amount and be placed for certain in the best group.

Indeed the expected payoff for player *i* playing $\alpha _h$ is at most:

5 $$\begin{aligned} E_{i}\left( \alpha _{h}\right) <w\left( 1-\alpha _{h}\right) +Q{\mathcal {S}}w\alpha _{h}^{\gamma }\left( 1-\frac{\tilde{c_{1}}}{c_{1}}\right) +Qw\left[ \alpha _{h}^{\gamma }\tilde{c_{1}}+l^{\gamma }\left( {\mathcal {S}}-\tilde{c_{1}}\right) \right] \left( \frac{\tilde{c_{1}}}{c_{1}} \right) \end{aligned}$$

where *l* is the strategy played by an agent $j \in C_2$ and hence $l < \alpha _h$. $\square$

By deviating, player *i* would surely be placed in the best group, gaining

6 $$\begin{aligned} E_{i}\left( \alpha _{h}+\varepsilon \right) =w\left( 1-\alpha _{h}-\varepsilon \right) +Q{\mathcal {S}}w\alpha _{h}^{\gamma }+O\left( \varepsilon \right) . \end{aligned}$$

We have that $E_{i}\left( \alpha _{h}\right) < E_{i}\left( \alpha _{h}+\varepsilon \right)$ if

$$\begin{aligned} \varepsilon < Q\frac{\tilde{c_{1}}}{c_{1}}\left[ \left( {\mathcal {S}}-\tilde{c_{1}}\right) \left( \alpha _{h}^{\gamma }-l^{\gamma }\right) \right] \end{aligned}$$

that for a small enough $\varepsilon$ is true. Hence player *i* would be better off deviating to $\alpha _h + \varepsilon$. Consequently, $\alpha _i = 1 \; \forall i \; \in C_1$.

#### Lemma 5

If there are some strategies $\alpha _i$ s.t. $\alpha _i > {\bar{\alpha }}$, then there cannot be any player *j* contributing ${\bar{\alpha }}< \alpha _j < 1$.

#### Proof

Let us call *j* the player with the highest contribution after all the players contributing *h*; i.e. $j \in C_2$ and let us call his strategy $\alpha _l$.

If there are no ties regarding group membership, *j* could reduce his contribution to $\alpha _l - \varepsilon$ and remain in the same group.

If there are ties, *j* could increase his contribution by an arbitrary small amount and be sure to be grouped together with players belonging to $C_1$. Similarly to Lemma 4, it is possible to prove that for an arbitrary small $\varepsilon$ we have:

$$\begin{aligned} E\left( \alpha _l \right) < E \left( \alpha _l + \varepsilon \right) \end{aligned}$$

and hence that $\alpha _j$ cannot be an equilibrium strategy. $\square$

#### Lemma 6

If there are some strategies $\alpha _i$ s.t. $\alpha _i > {\bar{\alpha }}$, then the number of players playing the within group best response is smaller than the group size; i.e. $z<{\mathcal {S}}$.

#### Proof

From Lemma 3 we know that $\left( H \; mod \; {\mathcal {S}} \right) > 0$ and hence

$$\left( \left( N-H\right) \; mod \; {\mathcal {S}} \right) > 0$$.

If *z* were bigger than the group size, a player *i* contributing ${\bar{\alpha }}$ could increase his payoff by an arbitrary small amount and be placed with certainty in the group containing some players contributing *h*.

Hence there is a profitable deviation and $z \ge {\mathcal {S}}$ could not be a Nash Equilibrium. $\square$

From Lemmata 3–6 follows that if an equilibrium s.t. $\alpha _i > {\bar{\alpha }}$ for some *i* exists, then each player plays either ${\bar{\alpha }}$ or 1. Furthermore, the number of players playing the within group best response is smaller than the group size.

From the lemmata above we can derive under which condition the generalized voluntary contribution game has a Nash Equilibrium with high contributions level. Hence the existence of a highly efficient equilibrium depends on the marginal per capita rate of return, the number of players and the size of the groups.

#### Theorem 1

For values of $\gamma$ bigger equal than a threshold value $\bar{\gamma }\left( Q,N,{\mathcal {S}}\right)$, the generalized voluntary contribution game has Nash Equilibria in which $z<{\mathcal {S}}$ players contribute ${\bar{\alpha }}$ and all the others $N-z$ players contribute 1. These equilibria are in addition to the equilibrium where all players contribute ${\bar{\alpha }}$.

For $\gamma < \bar{\gamma }$, the only NE is that all players contribute the within group best response ${\bar{\alpha }}$.

#### Proof

For $N-z$ players contributing 1 to be a NE, we have to show that no full contributor has a profitable deviation to contribute ${\bar{\alpha }}$ and that no player contributing ${\bar{\alpha }}$ has an incentive to play 1. We write

7 $$\begin{aligned} E_{1}\left( 1\right)= & {} w\frac{{\mathcal {S}}-z}{N-z}Q\left[ \left( {\mathcal {S}}-z\right) +z{\bar{\alpha }}^{\gamma }\right] +\frac{N-{\mathcal {S}}}{N-z}Q{\mathcal {S}}w \end{aligned}$$

8 $$\begin{aligned} E_{1}\left( {\bar{\alpha }}\right)= & {} w\left( 1-{\bar{\alpha }}\right) +wQ\left[ {\mathcal {S}}-z-1+\left( z+1\right) {\bar{\alpha }}^{\gamma }\right] \end{aligned}$$

9 $$\begin{aligned} E_{{\bar{\alpha }}}\left( {\bar{\alpha }}\right)= & {} w\left( 1-{\bar{\alpha }}\right) +wQ\left[ {\mathcal {S}}-z+z{\bar{\alpha }}^{\gamma }\right] \end{aligned}$$

10 $$\begin{aligned} E_{{\bar{\alpha }}}\left( 1\right)= & {} w\frac{{\mathcal {S}}-z+1}{N-z+1}Q\left[ \left( {\mathcal {S}}-z+1\right) +\left( z-1\right) {\bar{\alpha }}^{\gamma }\right] +\frac{N-{\mathcal {S}}}{N-z+1}Q{\mathcal {S}}w \end{aligned}$$

where we indicate with $E_{1}\left( \alpha \right)$ the payoff of a high contributor and with $E_{{\bar{\alpha }}}\left( \alpha \right)$ the payoff of a low contributor.

For the above to be a NE we have to have that (7) > (8) and (9) > (10).

The first condition is equivalent to:

11 $$\begin{aligned} z \ge \frac{\left( 1-{\bar{\alpha }}\right) N-QN\left( 1-{\bar{\alpha }}^{\gamma }\right) }{Q\left( 1-{\bar{\alpha }}^{\gamma }\right) \left[ N-1-{\mathcal {S}}\right] +1-{\bar{\alpha }}} \end{aligned}$$

The second condition leads to:

12 $$\begin{aligned} z \le 1 + \frac{\left( 1-{\bar{\alpha }}\right) N-QN\left( 1-{\bar{\alpha }}^{\gamma }\right) }{Q\left( 1-{\bar{\alpha }}^{\gamma }\right) \left[ N-1-{\mathcal {S}}\right] +1-{\bar{\alpha }}} \end{aligned}$$

However, it is important to remember that *z* needs to be smaller than the size of the groups.

Hence, we have that for values of the exponent $\gamma$ such that (11) is at most ${\mathcal {S}}-1$, the generalized voluntary contribution game has nearly efficient Nash Equilibria. For values of $\gamma$ s.t. (11) is bigger than ${\mathcal {S}}-1$ the only equilibrium is the one where all players play the within group best response. We call $\bar{\gamma }$ the value of $\gamma$ such that eq. (11)$={\mathcal {S}}-1$. $\square$

Hence we obtain the existence of a nearly-efficient high equilibrium depends on the marginal per capita rate of return, the number of players and the size of the groups.

### NE for Heterogeneous Players

Here we show that the near efficient Nash Equilibrium cannot exist for heterogeneous players. Furthermore, we derive under which condition the generalized voluntary contribution game has a pure strategy NE.

In the following we prove the lemmata necessary to derive the equilibrium.

#### Lemma 7

In case of full heterogeneity^11^ the assumptions of Lemma 3 cannot be satisfied.

#### Proof

Let’s assume that they are and show that this cannot be a NE. We call *k* the player with the lowest $w_i$ belonging to $C_1$.

We have two possibilities: $\left( a \right) s_k = w_k$ and $\left( b \right) s_k < w_k$

- $$\left( a \right) $$:
  Let’s take a player *j* s.t. $$j \in C_1 $$ and $$j \ne k$$. When playing $$s_j = s^1 $$, he has an expected payoff of at most (because in the mixed group there could also be players of classes lower than 2):
  13 $$\begin{aligned} E\left( j\right) \le w_{j}-s^{1}+Qs^{1}{\mathcal {S}}\cdot \left( 1-\frac{\widetilde{c}_{1}}{c_{1}}\right) +Q\left[ s^{1}\widetilde{c}_{1}+s^{2}\left( {\mathcal {S}}-\widetilde{c}_{1}\right) \right] \cdot \frac{\widetilde{c}_{1}}{c_{1}} \end{aligned}$$
  where $\frac{\widetilde{c}_{1}}{c_{1}}$ is the probability that agent *j* ends up in the group where not all agents belong to $C_1$.
  But agent *j* could play $\alpha _\varepsilon$ s.t. $s_j = s^1 + \varepsilon$, being guaranteed to end up in the first group and thus having an expected payoff of
  $$\begin{aligned} E_{\varepsilon }\left( j\right) =w_{j}-s^{1}-\varepsilon +Q\left( {\mathcal {S}}-1\right) s^{1}+Q\left( s^{1}+\varepsilon \right) \end{aligned}$$
  But if $\varepsilon <{\mathcal {S}}s^{1}\left( {\mathcal {S}}-\widetilde{c}_{1}\right) \frac{\widetilde{c}_{1}}{c_{1}}\left( 1-\frac{s_{2}}{s_{1}}\right)$ we have that $E_{\varepsilon }\left( j\right) > E\left( j\right)$.^12^ Hence there is a profitable deviation for agent *j*; so there can’t be a NE.
- $$\left( b \right) $$:
  The same as $\left( a \right)$, except that now even for *k* it is profitable to deviate to $s_k = s^1 + \varepsilon$.

Hence for heterogeneous players, it is impossible to maintain the conditions under which nearly efficient equilibrium was possible. $\square$

#### Lemma 8

In case of full heterogeneity of endowments $w_i$, with all $w_i$ having the same order of magnitude, $\alpha _i = {\bar{\alpha }} \; \forall i$ is not a Nash Equilibrium.

#### Proof

If all players were playing ${\bar{\alpha }}$, they would be ranked based on their endowments. Hence, a player *i* with the biggest endowment $w_i$ smaller than the biggest ${\mathcal {S}}$ endowments would have a profitable deviation by playing $\alpha _i = {\bar{\alpha }} + \varepsilon$ and be assigned to the best group.

If the endowments are such that $w_{i+1} > {\bar{\alpha }}w_i\; \forall i,$ 13 then there are no profitable deviations and $\alpha _i = {\bar{\alpha }} \; \forall i$ is a Nash Equilibrium. $\square$

From the above lemmas we can derive the following theorem:

#### Theorem 2

In case of full heterogeneity of endowments $w_i$, with all $w_i$ having the same order of magnitude, for $\gamma \ge 1$, the generalized voluntary contribution game has as only equilibrium non-contribution by all. For $0< \gamma < 1$, the game has no pure strategy NE and hence it has a mixed strategy Nash Equilibrium.

#### Proof

From Lemma 7 we know that the nearly efficient NE cannot exist for heterogeneous players. Furthermore, we can prove as in Lemma 5 that there can be no pure strategies such that ${\bar{\alpha }}< \alpha _i < 1$ for any player *i*.

From Lemma 1 we know that for $\gamma \ge 1$ the within group best response is to play $\alpha _i = 0 \; \forall i$. This can be an equilibrium and hence for values of $\gamma$ bigger than 1 there is a unique pure strategy NE for the generalized voluntary contribution game.

For $\gamma < 1$, however, the within group best response is to play $\alpha _i = {\bar{\alpha }}$. But Lemma 8 shows that this cannot be an equilibrium of the game (if the values of the endowment don’t differ too much). Hence for $\gamma < 1$ there are no pure strategy NE and thus there has to exist a mixed strategy Nash Equilibrium. $\square$

## Footnotes

1 Note that this kind of mechanism differs crucially from other contribution-inducing mechanisms such as ‘punishment’ (Fehr and Gächter 2000; Perc 2011) as no payoff additions, subtractions or transfer between individuals take place.

2
**Definition: **Efficiency is defined as the gain from the game relative to the initial endowment:

$$\begin{aligned} { Efficiency}=\frac{\sum _{i=1}^{N}\phi _{i}-\sum _{i=1}^{N}w_{i}}{\sum _{i=1}^{N}w_{i}}. \end{aligned}$$

3 Different initial starting conditions have been explored and they have been observed to have no effect on the final outcome of the simulation.

4 Inertia was added to ensure the convergence to the pure-strategy Nash equilibrium (if existing). Without inertia, the best-response dynamics could oscillate around such an equilibrium.

5 The interval $\left[ 0,1 \right]$ is discretized for computational purposes.

6 i.e. people contributing only ${\bar{\alpha }}$.

7 All players contributing ${\bar{\alpha }}$ is always an equilibrium for homogeneous players. The proof proceeds like in the linear case: it does not make sense to be the only player contributing more than that because this would only make the player’s groupmates better off at the player’s expense. Contributing less than ${\bar{\alpha }}$ is never beneficial, not even with random re-matching of groups, because of the non-linearity of the payoff function. See the “Appendix” for more details.

8 Here we use payoff dominant in the sense of Harsanyi and Selten (1988), Harsanyi (1995). The nearly efficient equilibrium can be shown to be ex-ante payoff dominant (Gunnthorsdottir et al. 2010a).

9 Note that there is no continuous convergence from the heterogeneous to the homogeneous case. This is a direct consequence of the fact that, as discussed before, as soon as heterogeneous endowments are introduced, there cannot be an equilibrium where more than one player contributes the same positive percentage of his endowment. This makes the existence of a mixed group impossible. See Lemma 7 in the “Appendix”.
