# Supplementary figures and images for: Assortative Matching with Inequality in Voluntary Contribution Games
Source: Comput Econ. 2017 Nov 18;52(3):1029–43. doi: 10.1007/s10614-017-9774-5 (PMC7444366; doi:10.1007/s10614-017-9774-5)

Efficiency loss in case of heterogenous players for mpcr= 0.4

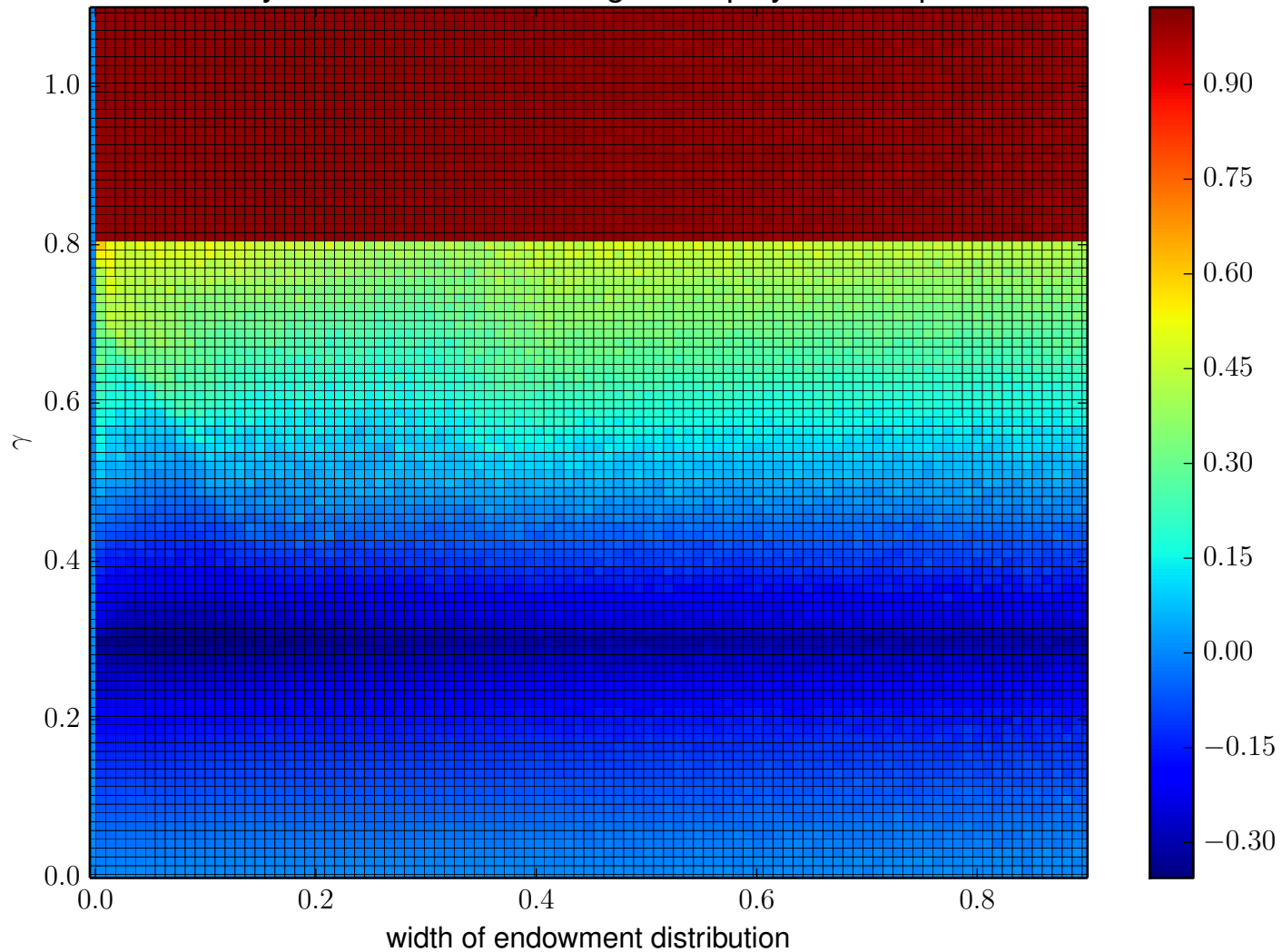

Supplement: Supplementary file 1 — Supplementary material 1 (pdf 232 KB) [file 10614_2017_9774_MOESM1_ESM.pdf]

Efficiency loss in case of heterogenous players for mpcr= 0.6

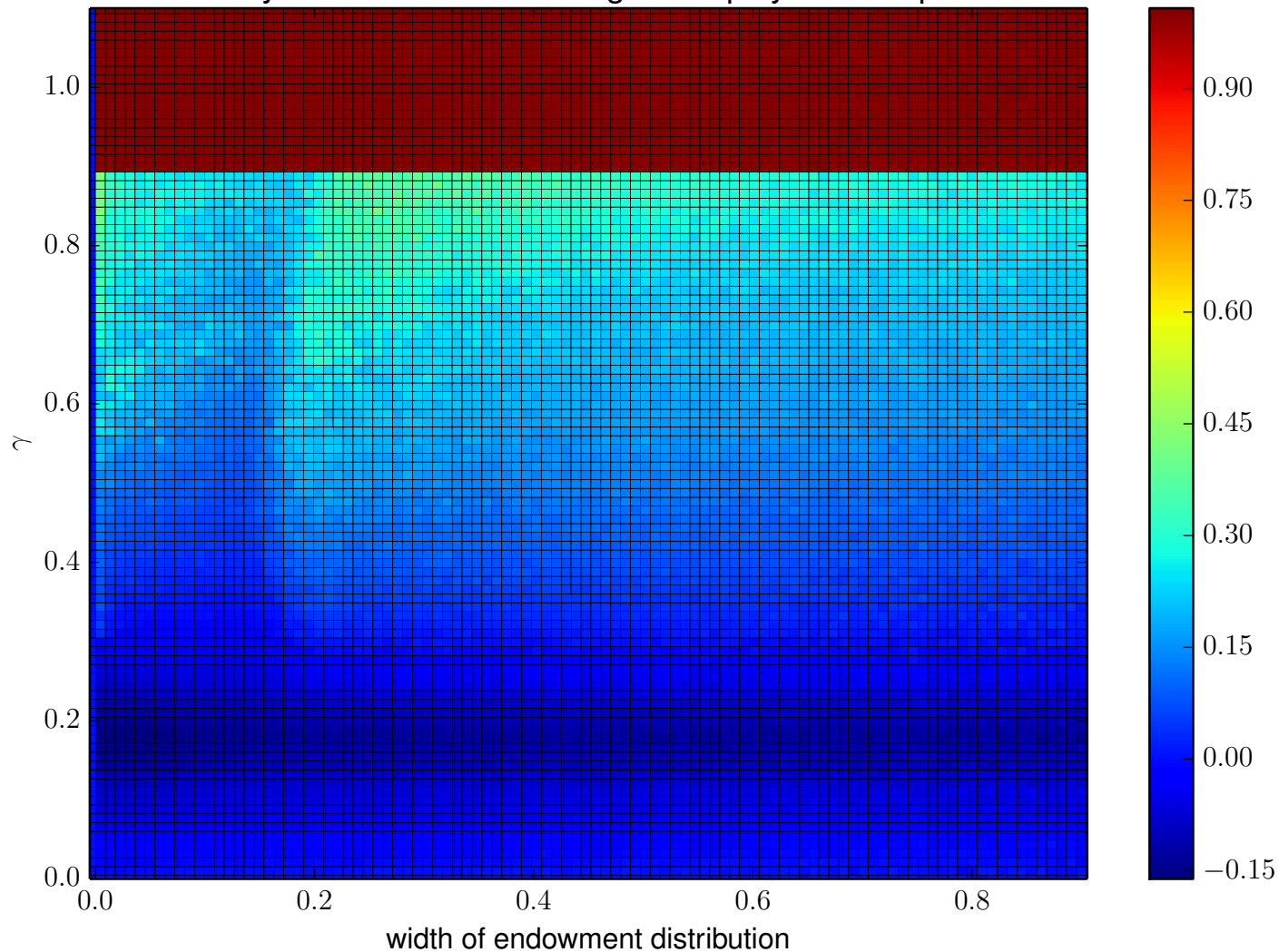

Supplement: Supplementary file 2 — Supplementary material 2 (pdf 231 KB) [file 10614_2017_9774_MOESM2_ESM.pdf]

Efficiency loss in case of heterogenous players for mpcr= 0.8

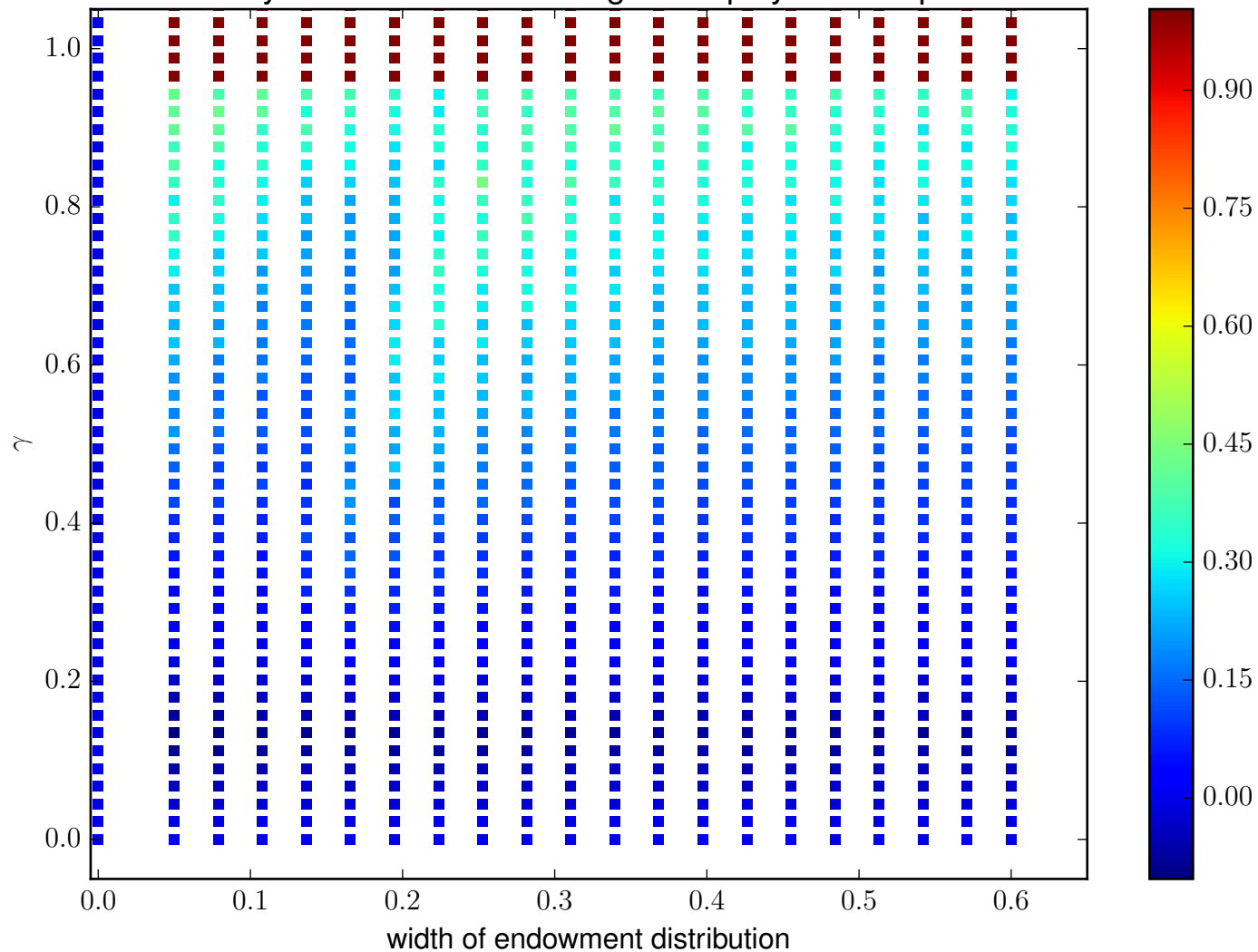

Supplement: Supplementary file 3 — Supplementary material 3 (pdf 118 KB) [file 10614_2017_9774_MOESM3_ESM.pdf]
